# Supplementary material for: HERON: A Novel Tool Enables Identification of Long, Weakly Enriched Genomic Domains in ChIP-seq Data
Source: Int J Mol Sci. 2021 Jul 29;22(15):8123. doi: 10.3390/ijms22158123 (PMC8348143; doi:10.3390/ijms22158123)
Supplement: Supplementary file 1 [file ijms-22-08123-s001.zip › ijms-1308802-SI.pdf]

Here we present supplementary data in two figures. The first one shows the comparison of different peakcallers performance in the setting where we would advise to use other tools than HERON (i.e., high signal-to-noise ratio and short peaks). The second one shows a variant of Figure 3 that is using sensitivity and specificity instead of TPR and FDR measures.

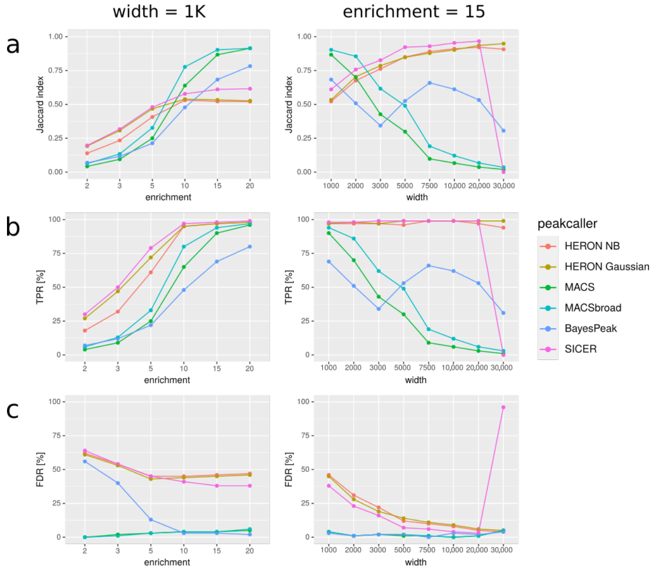

Figure 1: Comparison of peakcallers on simulated data for two different settings. In the first column, all the simulated peaks are 1K bp long, and simulated average enrichment varies. In the second column, average enrichment on peaks is constant and equal 15 and the peak length varies. In the rows three measures are shown: (a) Jaccard index: |predicted * real| / |predicted + real|, (b) TPR: |predicted * real| / |real|, and (c) FDR: |predicted \ real| / |predicted|; where: |predicted| - summaric size of regions predicted as peaks; |real| - summaric size of real peaks, i.e., simulated ones that we wanted to discover; A*B - intersection; A+B - union; A \ B - difference (A and not B).

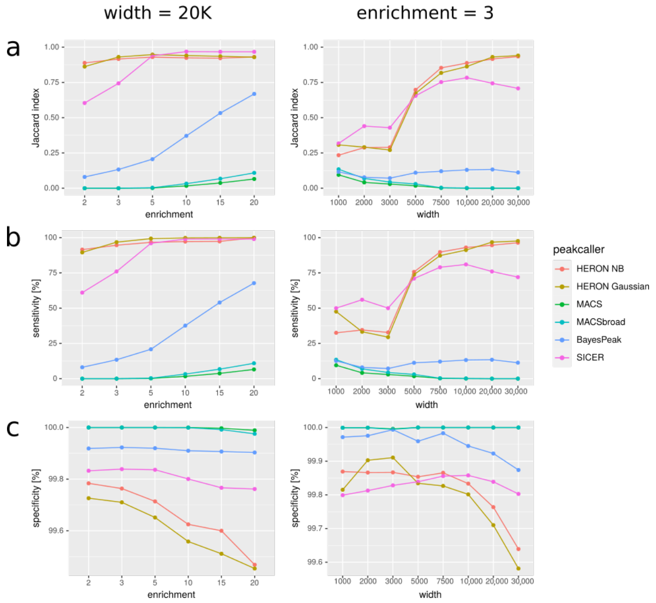

Figure 2: A version of Figure 3 presenting specificity instead of FDR. Please note the re-scaled Y axis to elucidate relatively small differences in overall high values of specificity.
